# Correlations of caregiver burden and musculoskeletal disorders with physical and psychosocial outcomes in palliative caregivers

**DOI:** 10.1007/s11845-026-04300-7

**Published:** 2026-03-24

**Authors:** Rabia Seva Ozkan, Sinem Suner-Keklik, Gamze Cobanoglu

**Affiliations:** 1 Sivas Cumhuriyet University Faculty of Health Sciences, Department of Physiotherapy and Rehabilitation, Sivas, Türkiye; 2 Gazi University Faculty of Health Sciences, Department of Physiotherapy and Rehabilitation, Ankara, Türkiye

**Keywords:** Caregiver burden, Pain, Posture, Quality of life

## Abstract

**Background:**

The increasing care burden associated with prolonged illness can lead to significant physical and emotional health problems in caregivers. Therefore, it is important to examine the effects of the caregiving burden experienced by palliative care providers on their physical and psychological well-being.

**Aims:**

This study was conducted to examine the relationship between the burden of care and musculoskeletal disorders in individuals providing palliative care and their physical activity level, posture, mood levels, and quality of life.

**Methods:**

The study included 56 caregivers (39 women, 17 men, age: 51.11 ± 12.38 years). Caregiver burden was measured using the Zarit Caregiver Burden Scale, musculoskeletal disorders using the Cornell Musculoskeletal Disorder Questionnaire (CMDQ) and physical activity level using the International Physical Activity Questionnaire Short Form. Posture was evaluated using the New York Posture Rating Chart, depression–anxiety–stress levels using the Depression Anxiety Stress Scale Turkish Short Form and quality of life using the 12-Item Short Form Questionnaire.

**Results:**

In the study, the burden of care was weakly negatively correlated with physical activity level (*r*=-0.268), posture (*r*=-0.398) and a moderate negative correlation with the physical component of quality of life (*r*=-0.421), (*p* < 0.05). It was also moderately positively correlated with anxiety (*r* = 0.568), depression (*r* = 0.609), and stress (*r* = 0.539), (*p* < 0.05). Spinal CMDQ scores were negatively correlated with posture (*r*=-0.339) and showed weak to moderate positive correlations with anxiety (*r* = 0.377), depression (*r* = 0.365), and stress (*r* = 0.410), (*p* < 0.05). Upper extremity CMDQ scores were weakly positively correlated with anxiety (*r* = 0.270), while lower extremity scores were negatively correlated with posture (*r*=-0.384) and the physical component of quality of life (*r*=-0.338), (*p* < 0.05).

**Conclusion:**

The palliative care process carries significant risk factors at both the physical and psychological levels. Therefore, it has been concluded that caregivers need to be assessed multidimensionally in future studies.

## Introduction

The global population is undergoing a rapid aging process, resulting in an increasing number of individuals reaching advanced ages. Concurrently, the prevalence of chronic diseases and cancer is rising, which leads to diverse care requirements during the end-of-life period [1, 2]. This growing demand underscores the importance of palliative care services in preserving quality of life and ensuring effective symptom control [2]. The World Health Organization defines palliative care as a holistic approach that seeks to enhance the quality of life of patients and their families confronting life-threatening conditions by addressing physical, psychosocial, and spiritual needs in an integrated manner [3].

Individuals providing palliative care are often described as “hidden patients”. The physically and emotionally demanding tasks they undertake may have significant adverse effects on both their physical and mental health [4]. The increasing care burden associated with prolonged illness can lead to significant physical health problems in caregivers [5, 6]. Particularly, repetitive and physically demanding daily activities, along with constant bending and lifting, may contribute to the development of musculoskeletal disorders in various parts of the body [7]. In addition, studies have reported that individuals providing palliative care experience caregiving-specific burdens and distress, as well as clinical levels of depression and anxiety [8, 9]. Depressive symptoms and anxiety among caregivers have been shown to compromise the immune system, increase the risk of illness, and negatively affect their ability to maintain personal health and overall quality of life [10].

In light of this information, it is important to examine how the care burden experienced by individuals providing palliative care, and the musculoskeletal disorders that may develop as a result of caregiving, affect their physical activity levels, postural alignment, emotional states, and quality of life. The existing literature has largely addressed these variables independently [6, 11, 12], and it is evident that the relationships between care burden and musculoskeletal disorders on one hand, and postural changes and physical activity levels on the other, have not been sufficiently investigated. In this context, the present study aims to evaluate the relationships comprehensively between care burden, musculoskeletal disorders, physical activity level, posture, mood, and quality of life among individuals providing palliative care. This study seeks to develop a holistic perspective on the health status of caregivers and to identify potential areas for intervention.

## Method

The necessary ethical approval for this study was obtained from the Non-Interventional Ethics Committee of Sivas Cumhuriyet University (date: 04/18/2024, number: 2024/04–28). The study was conducted in accordance with the principles of the Declaration of Helsinki and the Good Clinical Practice guidelines. Before starting the study, the content of the study was explained to the participants, and they were asked to sign an ‘Informed Consent Form’ to confirm their voluntary participation.

### Participants

Fifty-six individuals who met the inclusion criteria and who provided care to palliative care patients hospitalized at the Palliative Care Unit of Sivas Numune Hospital between June 1, 2024, and December 1, 2024, were included in this cross-sectional study. The study included individuals aged 18–65 who were first-degree relatives or regular caregivers of patients who had been hospitalized in the palliative care unit for at least one week, who could read and write Turkish and communicate, and who agreed to participate in the study voluntarily. Individuals with a diagnosed musculoskeletal disorder, a chronic health problem directly affecting posture and pain levels due to a neurological disorder or serious orthopedic problem, a history of psychiatric illness or cognitive impairment (to a degree that would prevent participation in the study), or who had undergone surgery within the last 6 months were excluded from the study.

## Procedure

Participants’ sociodemographic data, such as age, height, body weight, education level, and employment status, were recorded. In the study, the care burden was measured using the Zarit Caregiver Burden Scale, musculoskeletal disorders using the Cornell Musculoskeletal Disorder Questionnaire (CMDQ), physical activity level using the International Physical Activity Questionnaire Short Form, posture using the New York Posture Rating Chart (NYPRC), depression, anxiety, and stress levels were assessed using the Depression Anxiety Stress Scale Short Form (DASS-21), and quality of life was assessed using the 12-item Short Form Survey (SF-12).

### Assessment of care burden

The care burden was assessed using the Zarit Caregiver Burden Scale. Adapted into Turkish by İnci et al. in 2006, this scale was developed to measure the impact of caregiving on the caregiver’s life and consists of 22 questions [13]. The scale, which uses a Likert-type assessment, has a 5-point scale ranging from ‘0 = never’ to ‘4 = almost always’. A minimum of 0 and a maximum of 88 points can be obtained on the scale. A high scale score indicates an increase in the care burden [14].

### Assessment of musculoskeletal disorder severity

The Cornell Musculoskeletal Disorders Questionnaire, which measures the severity and frequency of pain and discomfort in the body, was used to determine the level of musculoskeletal disorders in individuals. The validity and reliability of the Turkish version of the scale have been determined by Erdinç and colleagues [15]. The questionnaire inquires about the severity, frequency, and impact on work of discomfort experienced in 19 different body regions over the past week; participants are asked to select the appropriate option for each item. The total score for each body region is calculated by multiplying the scores corresponding to these three components (frequency × severity × impact on work), and the total scale score is obtained by summing the scores for all body regions [15]. In our study, to ensure that the results are more understandable and interpretable, scale scores were analyzed under three main subscales covering the spine, upper extremity, and lower extremity regions, in parallel with a similar approach previously reported in the literature [16, 17]. In this context, higher scores correspond to a higher level of musculoskeletal disorders in the relevant region [17].

### **Assessment of physical activity level**

Individuals’ physical activity levels were assessed using the ‘International Physical Activity Questionnaire Short Form’. The short form includes seven questions about walking, time spent on moderate and vigorous activities, and frequency of activities over the past 7 days. Durations are multiplied by metabolic equivalents available on the scale for each activity. Based on their total physical activity level, individuals are classified into three levels: “inactive” (< 600 MET-min/week), “minimally active” (≥ 600 MET-min/week), and “active” (≥ 3000 MET-min/week) [18].

### Posture assessment

Individuals’ postures were assessed using the New York Posture Rating Chart (NYPRC). In this grading system, the person is observed from the side and front while standing upright, and points are awarded based on postural changes that may occur in 13 different parts of the body. If the postural structure in the examined area is correct, 5 points are given; if there is a moderate impairment, 3 points are given; if a severe impairment is observed, 1 point is given. At the end of the assessment, a score between 13 and 65 points is obtained. A final score of 45 points or above is classified as ‘very good’, a score between 40 and 44 points is classified as ‘good’, a score between 30 and 39 points is classified as ‘moderate’, a score between 20 and 29 points is classified as ‘poor’, and a score of 19 points or below is classified as ‘bad’ posture [19].

### Assessment of depression, anxiety, and stress

The ‘Depression Anxiety Stress Scale Turkish Short Form (DASS-21)’ was used to assess individuals’ levels of depression, anxiety, and stress. The Turkish adaptation of the 21-item scale was carried out by Yılmaz and colleagues under the name DASS-21. It is a 4-point Likert-type self-assessment form. The subscales of the scale consist of 7 items for depression, 7 items for anxiety, and 7 items for stress [20]. Each item in the subscale is scored between 0 (never) and 3 (very often), and the total score of the 7 items is multiplied by 2. The possible score range for each subscale is 0–42. High scores on each subscale indicate that the related psychological symptoms are experienced more severely [21].

### **Assessment of quality of life**

Individuals’ quality of life was assessed using the 12-item Short Form Questionnaire (SF-12). The scale consists of 12 selected questions from the SF-36 Questionnaire. The questionnaire, whose validity and reliability in Turkish were established by Soylu and Kütük, consists of eight subscales. It assesses an individual’s overall health status in two basic dimensions: Physical Component and Mental Component. Scores between 0 and 100 can be obtained from the scale. High scores indicate a high quality of life [22].

### Statistical analysis

Statistical analysis was conducted using IBM SPSS Statistics Version 26.0. Descriptive information was presented as mean (X) and standard deviation (SD). The normal distribution of the data was determined using visual (histogram and probability graphs) and analytical methods (Kolmogorov-Smirnov test). The correlation was evaluated using Pearson’s correlation. According to Pearson’s correlation coefficient (r), the degree of the relationship was categorized as: ≤ 0.10 negligible/very weak, 0.10–0.39 weak, 0.40–0.69 moderate, 0.70–0.89 strong, and ≥ 0.90 very strong correlation [23]. *p* < 0.05 was considered as statistically significant.

## Results

The post hoc power analysis for the study was calculated using the G*Power 3.1.9.6 program. Using the research data with a total sample size of 56, the correlation value of the study was found to be 0.609 (relationship between care burden and depression), and the power of the study (1-β) was calculated as 0.99 with a 5% error margin (α = 0.05) for the correlation analysis.

The demographic and descriptive characteristics of the participants are presented in Tables 1 and 2.

**Table 1:** Demographic information of participants

Characteristic		Participants (*n* = 56) (Mean ± SD)
Age (years)		51.11 ± 12.38
Weight (kg)		75.54 ± 13.25
Height (cm)		162.96 ± 8.5
BMI (kg/m^2^)		28.55 ± 5.21
		**n (%)**
Gender	Female	39 (69.6%)
	Male	17 (30.4%)
Marital status	Single	7 (12.5%)
	Married	45 (80.4%)
	Divorced or widowed	4 (7.1%)
Education	Less than high school graduate	37 (66.1%)
	High school graduate	10 (17.8%)
	College graduate	9 (16.1%)
Employment status	Employed	11 (19.6%)
	Not employed	45 (80.4%)

*SD* standard deviation, *BMI* Body Mass Index

**Table 2:** Mean scores and categorical classifications of assessment tools

		(Mean ± SD)	n (%)
Zarit CBS	**No or very light burden**	47.6 ± 15.96	0 (0%)
	**Mild burden**		19 (33.9%)
	**Moderate burden**		25 (44.6%)
	**Severe burden**		12 (21.4%)
CMDQ	**Spine**	33.68 ± 50.98	
	**Upper extremity**	25.48 ± 53.45	
	**Lower extremity**	33.26 ± 60.13	
IPAQ (MET-min/week)	**Light activity (Walking)**	723.94 ± 511.18	
	**Moderate activity**	674.29 ± 493.38	
	**Vigorous activity**	2080.00 ± 1321.82	
	**Total activity**	674.8 ± 830.39	
IPAQ (Categoric levels)	**Inactive**		31 (55.4%)
	**Minimally active**		24 (42.9%)
	**Active**		1 (1.8%)
NYPRC	**Bad**	43.8 ± 11.55	0 (0%)
	**Poor**		8 (14.3%)
	**Medium**		15 (26.8%)
	**Good**		3 (5.4%)
	**Very good**		30 (53.6%)
DASS-21	**Anxiety**	12.6 ± 8.43	
	**Depression**	15.6 ± 10.25	
	**Stress**	17.17 ± 9.63	
SF-12	**Physical component**	44.63 ± 10.63	
	**Mental component**	36.55 ± 9.37	
	**Total score**	81.19 ± 12.04	

*CBS* caregiver burden scale, *CMDQ* Cornell musculoskeletal disorder questionnaire, *IPAQ* international physical activity questionnaire, *NYPRC* New York posture rating chart, *DASS*-21 21-item depression anxiety stress scale

Caregiver burden was found to be weakly negatively correlated with posture and physical activity level, and moderately negatively correlated with the physical component of quality of life. Additionally, it was moderately positively correlated with anxiety, depression, and stress scores (*p* < 0.05, Table 3).

**Table 3:** Associations of caregiver burden and musculoskeletal disorders pain with posture, physical activity, mental health and quality of life

			IPAQ	NYPRC	DASS-21 Anxiety	DASS-21 Depression	DASS-21 Stress	SF-12 Physical	SF-12 Mental
Zarit CBS		**r**	-0.268*	-0.398*	0.568*	0.609*	0.539*	-0.421*	0.174
		**p**	**0.046**	**0.002**	**< 0.000**	**< 0.000**	**< 0.000**	**0.001**	0.200
CMDQ	Spine	**r**	-0.217	-0.339*	0.377*	0.365*	0.410*	-0.105	0.049
		**p**	0.108	**0.011**	**0.004**	**0.006**	**0.002**	0.442	0.720
	Upper Extremity	**r**	-0.082	-0.194	0.270*	0.170	0.106	0.039	0.089
		**p**	0.548	0.151	**0.045**	0.209	0.436	0.773	0.513
	Lower Extremity	**r**	-0.106	-0.384*	0.115	0.134	0.102	-0.338*	-0.037
		**p**	0.437	**0.003**	0.399	0.325	0.454	**0.011**	0.788

*p < 0.05, *CBS* caregiver burden scale,* CMDQ* Cornell musculoskeletal disorder questionnaire, *IPAQ* international physical activity questionnaire, *NYPRC*: New York posture rating chart, *DASS*-21: 21-item depression anxiety stress scale

The analysis revealed that scores obtained from the spinal subdimension of the CMDQ were negatively correlated with posture, while weak positive correlations were observed with anxiety and depression. Furthermore, spinal subdimension scores demonstrated a moderate positive correlation with stress levels (*p* < 0.05, Table 3).

Scores from the upper extremity subdimension of the CMDQ exhibited a weak positive correlation only with anxiety scores (*p* < 0.05, Table 3). Additionally, scores from the lower extremity subdimension were negatively correlated with posture and the physical component of quality of life (*p* < 0.05, Table 3).

## Discussion

This study aimed to examine the relationships between caregiving burden and musculoskeletal disorders among individuals providing palliative care, as well as their physical activity levels, posture, mood, and quality of life. The findings indicated that increased caregiving burden and musculoskeletal disorders among caregivers were associated with limited participation in physical activity and negative effects on posture. Such outcomes may lead to a deterioration in psychological well-being, symptoms of stress, anxiety, and depression, and a decline in quality of life.

Caregiving is a continuous process of providing assistance and support, most often by family members, to individuals requiring help with daily living activities due to chronic illness, advanced age, or dependency [10]. However, long-term and intensive caregiving responsibilities can limit the caregiver’s own life over time, leading to physical, psychological, social, and economic difficulties, a situation defined in the literature as the burden of caregiving [24, 25]. In the literature, it has been reported that the increased burden of caregiving associated with prolonged physical exertion increases susceptibility to musculoskeletal problems and negatively affects functional levels [7, 26, 27]. Caregivers may be unable to maintain regular exercise and active lifestyle habits due to the intense time demands and physical fatigue associated with caregiving activities [28]. A study of caregivers of Chronic Obstructive Pulmonary Disease (COPD) patients showed a negative relationship between caregiving burden and physical activity levels. Hipólito et al. (2020) reported in their study that increased caregiving burden, along with emotional and financial burden, led to a significant decrease in physical activity levels, along with negative effects on personal life. In particular, it was stated that the prolongation of caregiving duration, along with increased caregiving burden and emotional stress, may have caused caregivers to withdraw from active life [29]. It was found that 33.9% of caregivers participating in our study experienced a mild level of caregiving burden, 44.6% experienced a moderate level, and 21.4% experienced a severe level. In our study, 55.4% of caregivers were physically inactive, 42.9% were minimally active, and 1.8% were active. These rates indicate that the majority of individuals included in the study experience a caregiving burden and that more than half are inactive. Our study showing that 55.4% of caregivers are not physically active is consistent with studies in the literature reporting high rates of physical inactivity. In particular, a study of caregivers of older adults reported a prevalence of physical inactivity of approximately 75%, indicating that the vast majority of caregivers do not engage in sufficient physical activity [30]. In another study in the context of palliative care, it has been noted that caregivers’ long-term care burden and social isolation may reduce their likelihood of being physically active and increase their sedentary lifestyles [31].

The weak negative correlation identified in our study between the caregiving burden and physical activity level supports findings in the literature. This finding suggests that as the caregiving burden increases, individuals tend to become less physically active, and that the caregiving process may make it difficult to maintain an active lifestyle. Furthermore, we believe that increased responsibilities may cause caregivers to neglect their own health, reducing their physical activity levels, which may have negative repercussions on their overall health. However, our study did not find a significant relationship between physical activity level and musculoskeletal disorders. This finding suggests that low physical activity levels may be related to time constraints, fatigue, and changes in priorities brought about by the caregiving burden rather than musculoskeletal complaints.

It has been reported that static positions repeatedly encountered in daily life by individuals providing long-term care services increase mechanical loading, and that this loading leads to chronic pain and discomfort in spinal segments over time. Chronic pain and discomfort in the musculoskeletal system also cause postural disorders [26, 32]. In a cross-sectional study examining musculoskeletal problems in caregivers of advanced cancer patients, 31.4% of family caregivers were found to have chronic thoracic spine pain, and 28.3% had chronic low back pain [32]. Similarly, a study by Karaman and Özdemir [33] examined the effects of back and neck pain, which developed in caregivers due to the burden of care, on posture and found that this pain dramatically impaired posture [33]. The literature specifically mentions that back and neck pain directly cause common postural changes such as thoracic kyphosis and cervical protraction [34–36]. In our study, it was determined that posture was negatively affected among caregivers with high levels of pain and musculoskeletal disorders. The negative correlation between the scores obtained from the CMDQ’s spine and lower extremity subscales and posture supports the view that postural impairments are closely related to musculoskeletal disorders. The increased care burden may have physically strained the individual, leading to musculoskeletal fatigue, muscle spasms, and discomfort. In particular, prolonged static positions and physically demanding tasks such as lifting or supporting the patient may have increased mechanical stress on the spine and surrounding muscles, causing pain and discomfort. This situation may cause changes in posture by disrupting the caregiver’s spinal alignment. Although our study found a significant correlation between spinal and lower extremity discomfort and posture, no similar relationship was observed with upper extremity discomfort. This suggests that upper extremity pain and discomfort may stem from different factors, such as repetitive movements, local trauma, or non-ergonomic use of the upper extremities, rather than impaired posture. Furthermore, the low prevalence of upper extremity disorders among the participants in our study may have contributed to the lack of a significant relationship with posture.

The psychological effects of caregiving responsibilities on the mental health of caregivers and the potential consequences thereof are fundamental issues that must always be addressed in terms of caregivers’ mental health. Current studies frequently report stress, anxiety, worry, depression, isolation, and anger as psychological consequences of caregiving [37]. In previous studies, depression and anxiety were among the most frequently reported psychological consequences, with a prevalence ranging from 52% to 94% among family caregivers. The severity and prevalence of these psychological problems can sometimes be even higher than those of the patients they care for [38]. Current evidence shows that family caregivers experience increased stress and psychosocial distress when they have to balance their professional careers with their domestic responsibilities [39]. In a study of caregivers of cancer patients, 83% of participants were assessed as being at risk in terms of care burden, with 30.7% falling into the severe burden category. Additionally, the burden of caregiving has been found to correlate positively with both depression and anxiety levels. The prevalence of these significant psychological outcomes is thought to stem from the physical and mental burdens caregivers face [38].

As in our study, another study using the Zarit Caregiver Burden Scale also showed that high caregiving burden is associated with increased severity of anxiety and depression symptoms [40]. According to our findings, caregiver burden shows a moderate positive correlation with anxiety, depression, and stress levels. This result suggests that the caregiving process involves not only physical but also intense psychological burdens, which can negatively affect caregivers’ mental health. The continuity of caregiving responsibilities and sometimes unpredictable demands may have challenged individuals’ capacity to cope with stress, leading to increased anxiety levels, emotional exhaustion, and susceptibility to depressive symptoms. Particularly in long-term and emotionally intense processes such as palliative care, the pressure on caregivers’ physical and psychological resilience may have increased feelings of exhaustion, leading to various limitations in daily functioning.

The literature frequently highlights the reciprocal interaction between pain and mood disorders. Some studies have reported that psychosocial stress can increase pain perception by causing changes in muscle tone [41, 42]. Meta-analysis results reveal that pain threshold is lowered and perceived pain intensity is increased in individuals with anxiety [43]. Findings regarding pain threshold in depressed individuals vary across studies, with some reporting a decrease and others an increase; however, the general view is that mood disorders can modulate pain perception [44]. Furthermore, it has been observed that as stress and anxiety levels increase, individuals’ pain tolerance decreases significantly [43, 45]. In our study, the fact that the scores obtained from the spine subscale of the CMDQ showed a weak positive correlation with anxiety and depression levels and a moderate positive correlation with stress levels reveals that spinal pain is closely related not only to physical but also to psychological factors. The moderate positive correlation identified with stress in the lower back dimension supports the “stress-pain cycle” mechanism, whereby stress increases pain perception through the central nervous system. In the upper extremity subscale, the presence of only a weak positive correlation with anxiety levels suggests that anxiety may exacerbate upper extremity symptoms through muscle tension and avoidance of movement. In contrast, the absence of a significant relationship with psychological variables in the lower extremity subscale suggests that the etiology of pain in this region may be more likely to stem from local biomechanical factors.

The literature indicates that long-term and high-intensity caregiving processes may increase somatic complaints such as physical exhaustion, musculoskeletal disorders, fatigue, and sleep disorders in individuals, thereby reducing their quality of life [46, 47]. In a study examining the relationship between care burden and quality of life in caregivers of hemodialysis patients, it was found that 37.4% of caregivers experienced high and very high levels of care burden, while 42.7% experienced moderate levels of care burden, and it was stated that their quality of life was dramatically negatively affected due to the care burden [48]. Another study examined the relationship between caregiver burden and quality of life in caregivers of cancer patients and reported a significant decrease in caregivers’ quality of life as caregiver burden increased [49]. Our study also found a moderate negative relationship between caregiver burden and the physical component of quality of life. This relationship may be related to caregivers neglecting their own health due to increased responsibilities, as well as factors such as stress, sleep disturbance, and decreased physical activity caused by the intensive care process. These multiple effects may have caused a decline in the physical component of quality of life by negatively affecting physical capacity and well-being.

This study has several limitations. We did not select caregivers from those caring for individuals with specific illnesses. This can be considered a limitation of our study, as the physical and psychological burden of each illness differs for both the individual and those around them. Furthermore, the fact that the majority of individuals included in the assessment share similar sociocultural characteristics may make it difficult to generalize the results to different cultures or demographic groups. Furthermore, since the study was conducted at a single center, the generalizability of the findings to caregivers in different healthcare settings or regions may be limited. Since the assessments were conducted at a single point in time, it was not possible to examine the course of possible changes in the care burden and psychosocial status over time. Long-term studies involving individuals with different sociocultural characteristics are needed. Furthermore, the physical assessments in our study are based on questionnaires and observational measurements. Additional studies with objective assessments are needed to more clearly demonstrate the effects on individuals providing palliative care. In addition, including carers who have been providing care for longer periods of time could have made the results clearer.

## Conclusion

This study revealed the relationship between the burden of care and musculoskeletal disorders in individuals providing palliative care and their physical activity level, postural alignment, mood levels, and quality of life. It was found that an increase in the burden of care and a rise in the level of musculoskeletal disorders create interrelated chain effects, such as a decrease in physical activity levels, deterioration of posture, increased anxiety, stress, and depression, and a significant decline in quality of life. It has been suggested that the physical and emotional burden experienced by caregivers creates a vicious cycle that can pose increasing risks to both physical and psychological health. Clinically, it should not be forgotten that caregivers are a group that needs protection not only for the sake of the patient but also for their own health. Therefore, conducting regular health screenings for individuals providing palliative care, evaluating posture analyses and providing guidance based on the results, and offering informative training can increase awareness among individuals and lead to positive outcomes related to their overall health. Additionally, encouraging caregivers to exercise regularly, strengthening approaches to stress management and psychosocial support mechanisms, and reducing factors associated with the negative effects of the caregiving burden can contribute to maintaining quality of life. These results indicate that the palliative care process involves significant risk factors at both the physical and psychological levels, and therefore, future studies should involve a multidimensional assessment of caregivers.
